# Acquired Resistance to Antituberculosis Drugs in England, Wales, and Northern Ireland, 2000–2015

**DOI:** 10.3201/eid2403.171362

**Published:** 2018-03

**Authors:** Miranda G. Loutet, Jennifer A. Davidson, Tim Brown, Martin Dedicoat, H. Lucy Thomas, Maeve K. Lalor

**Affiliations:** Public Health England, London, UK (M.G. Loutet, J.A. Davidson, T. Brown, H.L. Thomas, M.K. Lalor);; Heart of England NHS Foundation Trust, Birmingham, UK (M. Dedicoat);; University College London, London (M.K. Lalor)

**Keywords:** tuberculosis and other mycobacteria, TB, drug resistance, acquired resistance, antimicrobial resistance, United Kingdom, England, Wales, Northern Ireland, bacteria

## Abstract

Among tuberculosis (TB) patients, acquired resistance to anti-TB drugs represents a failure in the treatment pathway. To improve diagnosis and care for patients with drug-resistant TB, we examined the epidemiology and risk factors associated with acquired drug resistance during 2000–2015 among TB patients in England, Wales, and Northern Ireland. We found acquired resistance in 0.2% (158/67,710) of patients with culture-confirmed TB. Using multivariate logistic regression, we identified the following factors associated with acquired drug resistance: having pulmonary disease; initial resistance to isoniazid, rifampin, or both; a previous TB episode; and being born in China or South Africa. Treatment outcomes were worse for patients with than without acquired resistance. Although acquired resistance is rare in the study area, certain patient groups are at higher risk. Identifying these patients and ensuring that adequate resources are available for treatment may prevent acquisition of resistance, thereby limiting transmission of drug-resistant strains of mycobacteria.

In 2015, of an estimated 10.4 million incident tuberculosis (TB) cases worldwide, 4.6% were multidrug resistant (MDR) (*1*). MDR TB is a major challenge for TB control, and strategies to reduce it underpin the 3 pillars of the World Health Organization End TB Strategy (*1*,*2*). The main challenges are preventing acquisition of drug resistance and ensuring early detection and appropriate treatment to prevent further transmission of drug-resistant TB. As a result, in the Collaborative TB Strategy for England 2015–2020, reducing drug-resistant TB is a key area of action for ensuring that adequate resources needed to prevent and treat MDR TB exist (*3*).

In the United Kingdom, the proportion of MDR TB cases has remained stable over the past decade, accounting for 1.5% of all culture-confirmed TB cases in 2015, similar to the proportions in other low-incidence countries, including the United States (1.2%) and France (1.7%) (*1*,*4*). Other drug-resistance profiles affect treatment and include isoniazid resistance without MDR TB (attributable to 5.6% of TB cases in the United Kingdom) and rifampin resistance without MDR TB (accounts for only 0.2% of TB cases) (*4*). In addition to the clinical challenges of long and complex treatment regimens often involving adverse reactions for patients with drug-resistant TB, social risk factors and concurrent conditions may complicate treatment and worsen outcomes (*5*).

Drug-resistant TB can arise in 2 ways: through transmission of a drug-resistant strain (primary drug resistance) or through acquisition of drug resistance (acquired drug resistance, in which a person is infected by a strain that is initially sensitive to a particular drug but resistance to that drug evolves later) (*6*,*7*). Acquired drug resistance can result from inadequate treatment, which may be caused by interruptions to receipt of the full drug regimen (*8*,*9*). Treatment interruption because of drug unavailability, poor adherence, or side effects contributes to insufficient dosing or treatment duration (*7*).

Acquired drug resistance has consequences for public health through the spread of drug-resistant TB, poorer health outcomes for patients, and cost to the healthcare system (*10*–*12*). Understanding the risk factors associated with acquired resistance could improve prevention measures. Our objective for this study was to describe the epidemiology of TB among patients in England, Wales, and Northern Ireland, in whom drug resistance was acquired while they were receiving treatment. We also examined the frequency and timing of acquired resistance, sociodemographic and clinical factors associated with acquired resistance, and treatment outcomes for these patients.

## Methods

### Study Population and Definitions

We conducted a retrospective cohort study of all patients with culture-confirmed TB (pulmonary and extrapulmonary-only disease) notified to the Enhanced TB Surveillance System in England, Wales, and Northern Ireland during 2000–2015 for whom initial drug-susceptibility testing (DST) results for at least isoniazid and rifampin were available. Acquired resistance was defined as a sensitive DST result for a drug on 1 isolate and a subsequent resistant DST result for the same drug on another. We excluded TB patients known to have not started treatment.

### Laboratory Methods and Data

The United Kingdom follows the global guidance that samples should be obtained for culture and DST at least every 1, 2, and 5 months, whereas samples from patients whose TB was initially MDR TB should be obtained every month (*13*–*15*). Samples are collected more often from patients who do not clinically respond to treatment as expected.

Samples from all presumptive TB patients are sent from hospital laboratories in England, Wales, and Northern Ireland to a *Mycobacterium* reference laboratory for speciation, phenotypic DST, and 24-loci MIRU-VNTR (mycobacterial interspersed repetitive unit–variable-number tandem repeat) strain typing (*16*). Results from culture-confirmed *M. tuberculosis* complex samples were routinely extracted from laboratory information management systems and imported into the Enhanced TB Surveillance System. The strain lineage was derived from the MIRU-VNTR strain types for cases notified during 2010–2015 (*17*). The reference laboratories routinely performed DST for first-line drugs (isoniazid, rifampin, ethambutol, and pyrazinamide) on all *M. tuberculosis* complex samples. DST for second-line injectables (amikacin, capreomycin, and kanamycin) and fluoroquinolones (ofloxacin, moxifloxacin, and ciprofloxacin) was performed on rifampin-resistant isolates or at the request of the treating clinician. DST results for ethambutol and pyrazinamide can vary (*18*), which was mitigated at the reference laboratory by repeat testing of isolates that initially showed resistance.

### Data Collection

Clinical and demographic data for TB patients were collected in the Enhanced TB Surveillance System and probabilistically matched to *M. tuberculosis* complex isolates (*5*,*19*). We analyzed individually the 8 countries of birth with the highest number of TB patients with acquired drug resistance. Data on patient social risk factors (current or history of drug misuse, alcohol misuse, homelessness, and imprisonment) were available for cases notified during 2010–2015. Treatment outcome was reported for cases notified during 2001–2013.

### Descriptive Analyses

We described cases with acquired resistance by year of notification. The proportion of TB patients in whom resistance to each drug was acquired was calculated from the number of TB patients for whom DST results for that drug were available. TB patients with acquired resistance were categorized according to their initial drug resistance profile (Table 1) and acquired drug resistance profile (Table 2) throughout the analysis. Each new identification of resistance in different cultures from the same patient was defined as an episode of acquired resistance.

**Table 1:** Initial drug resistance profiles used to categorize resistance to anti-TB drugs*

Initial drug resistance profile	Description of drug-resistance profile†
Drug sensitive	Initially sensitive to both isoniazid and rifampin
Isoniazid resistance without MDR TB	Initially resistant to isoniazid and sensitive to rifampin
Rifampin resistance without MDR TB	Initially resistant to rifampin and sensitive to isoniazid
MDR TB	Initially resistant to both isoniazid and rifampin

*MDR, multidrug-resistant; TB, tuberculosis. †These profiles refer specifically to resistance to isoniazid and/or rifampin, but resistance to other drugs may also be present.

**Table 2:** Description of acquired drug resistance profiles used to categorize resistance to anti-TB drugs*

Acquired drug resistance profile	Description of drug-resistance profile
TB patients who did not have MDR TB before or after acquiring resistance	Initially sensitive to isoniazid, rifampin, or both and may have acquired resistance to either isoniazid or rifampin but not both or may have acquired resistance to other anti-TB drug(s).
TB patients who acquired resistance that resulted in MDR TB	Initially sensitive to isoniazid, rifampin, or both and acquired resistance to isoniazid, rifampin, or both to have MDR TB.
Patients with initial MDR TB and acquired resistance to additional drugs	Initially resistant to at least isoniazid and rifampin and on subsequent culture acquired resistance to other anti-TB drug(s).

*MDR, multidrug-resistant; TB, tuberculosis.

The time to diagnosis of acquired resistance (referred to as acquiring drug resistance) was calculated from the treatment start date to the specimen collection date for the first DST result that showed evidence of acquired resistance. The time to diagnosis of acquired resistance was calculated for TB patients who acquired resistance after starting an appropriate treatment; therefore, we excluded from this section of analysis those patients who acquired resistance before starting an appropriate treatment or who did not have a known start date for appropriate treatment.

### Statistical Analyses

We compared clinical and demographic data among patients with any acquired resistance with those without acquired resistance by using univariable logistic regression to calculate odds ratios (ORs). We created a multivariable model by using a forward stepwise approach, including factors with p<0.5 from the univariable analysis and using data from the entire cohort (2000–2015) (model 1). We created a second multivariable model limiting the data to 2010–2015 because of the availability of social risk factors and strain type lineage data (model 2).

To account for the variability in resistance results for ethambutol and pyrazinamide, we performed a sensitivity analysis by excluding from logistic regression model 1 those TB patients who acquired resistance to only ethambutol and pyrazinamide. Because of low numbers and loss of power, we did not perform a sensitivity analysis on model 2.

We used χ^2^ tests to compare treatment outcomes among those with and without acquired drug resistance. All analyses were conducted by using Stata version 13.1 (StataCorp LLC, College Station, TX, USA).

## Results

**Figure 1:**
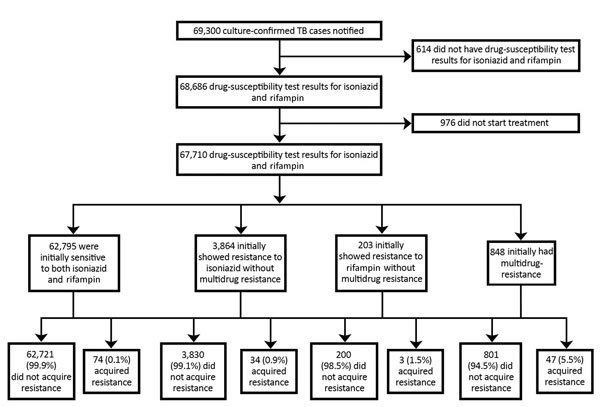
Process used to select tuberculosis (TB) patients with acquired drug resistance among all TB patients with culture-confirmed TB, England, Wales, and Northern Ireland, 2000–2015.

**Figure 2:**
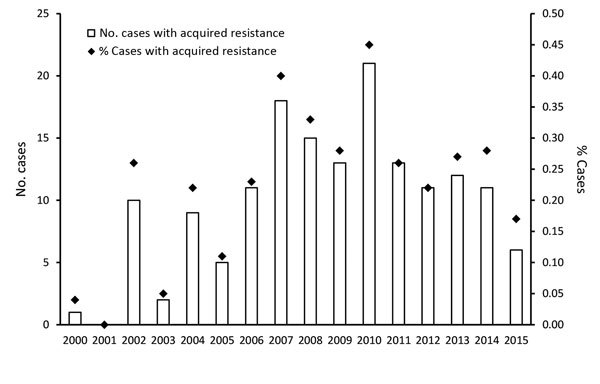
Number and proportion of tuberculosis patients with acquired drug resistance, by year, England, Wales, and Northern Ireland, 2000–2015.

During 2000–2015, a total of 69,300 culture-confirmed TB cases were notified in England, Wales, and Northern Ireland; among these, 99.1% (68,686/69,300) had DST results for at least isoniazid and rifampin (Figure 1). We excluded 976 TB patients known to have not started treatment. Overall, 0.2% (158/67,710) of culture-confirmed TB patients with DST results acquired drug resistance. The median number of TB patients with acquired resistance per year was 11 (range 0–21) (Figure 2). The number of TB patients with acquired resistance increased over time (p = 0.002).

**Figure 3:**
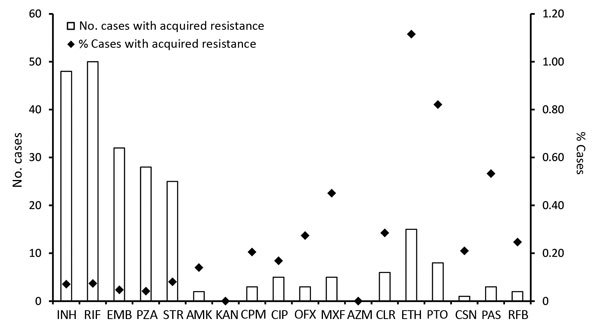
Number and proportion of tuberculosis patients with acquired drug resistance, by drug, England, Wales, and Northern Ireland, 2000–2015. AMK, amikacin; AZM, azithromycin; CIP, ciprofloxacin; CLR, clarithromycin; CPM, capreomycin; CSN, cycloserine; EMB, ethambutol; ETH, ethionamide; KAN, kanamycin; INH, isoniazid; MXF, moxifloxacin; OXF, ofloxacin; PAS, para-aminosalicylic acid (bacteriostatic); PTO, prothionamide; PZA, pyrazinamide; RIF, rifampin; RFB, rifabutin; STR, streptomycin.

Among patients tested for each drug, the highest numbers acquired resistance to rifampin (50/67,710), isoniazid (48/67,710), ethambutol (32/67,645), and pyrazinamide (28/67,061). The highest percentage of patients had acquired resistance to ethionamide (1.1%, 15/1,345) and prothionamide (0.8%, 15/1,345) (Figure 3).

### Initial Drug-Resistance Profile

Isolates from most TB patients with acquired resistance were initially sensitive to both isoniazid and rifampin (46.8%, 74/158) (Table 3). Resistance to additional drugs was acquired by 0.1% (74/62,795) of TB patients with isolates initially sensitive to isoniazid and rifampin, 0.9% (34/3,864) of TB patients with isoniazid resistance and without rifampin resistance, 1.5% (3/203) of TB patients with rifampicin resistance and without isoniazid resistance, and 5.5% (47/848) of MDR TB patients. 

**Table 3:** TB cases according to drug-resistance profile, England, Wales, and Northern Ireland, 2000–2015*

Initial drug-resistance profile, no. patients with acquired drug resistance	Acquired resistance and MDR TB developed later, no. (%)	Initial MDR TB with additional resistance acquired later, no. (%)	No MDR TB before or after acquiring resistance, no. (%)
Drug sensitive, n = 74	20 (27.0)	NA	54 (73.0)
Isoniazid resistant without MDR, n = 34	22 (64.7)	NA	12 (35.3)
Rifampin resistant without MDR, n = 3	2 (66.7)	NA	1 (33.3)
MDR TB, n = 47	NA	47 (100)	NA
Total, n = 158	44 (27.8)	47 (29.7)	67 (42.4)

*MDR, multidrug-resistant; NA, not applicable; TB, tuberculosis.

For 44 TB patients, acquired resistance resulted in MDR TB; among these patients, initial isolates from 20 were sensitive to both isoniazid and rifampin, 22 were resistant to isoniazid without MDR TB, and 2 were resistant to rifampin without MDR TB. A total of 67 TB patients acquired resistance without it being MDR TB (initial or acquired); the highest proportion was resistant to isoniazid (32.8%, 22/67), followed by streptomycin (22.4%, 15/67) and pyrazinamide (14.9%, 10/67).

### Episodes of Acquired Resistance

Resistance was acquired to 1–6 drugs; most (69.0%, 109/158) resistance was to only 1 drug, 19.6% (31/158) to 2 drugs, and 6.3% (10/158) to >3 drugs. Of those who acquired resistance to >2 drugs, 65.3% (32/49) acquired resistance in 1 episode, 28.6% (14/49) over 2 episodes, and 6.1% (3/49) over 3 episodes. The median time between the first and second episode for those who had at >2 episodes was 3.5 months (interquartile range [IQR] 2.4–11.9 months), and the median time between the second and third episode for those who had 3 repeat episodes was 5.3 months (IQR 4.7 months–2 years).

### Time between Starting Treatment and Acquiring Resistance

Ten MDR TB patients acquired resistance between starting an initial regimen and switching to the appropriate regimen for drug resistance. These patients acquired resistance to ethambutol (4), pyrazinamide (1), ethionamide (4), and prothionamide (1).

A total of 136 TB patients acquired resistance after starting an appropriate treatment regimen; median time between starting treatment and acquiring resistance to >1 drug or to the first drug was 3.4 months (IQR 1.5–8.3 months). Resistance was acquired by 12% (17/136) of patients 1 year after starting treatment (Table 4).

**Table 4:** Number and proportion of TB cases, by time between treatment initiation and drug resistance acquisition and by acquired drug resistance categories, England, Wales, and Northern Ireland, 2000–2015*

Time between treatment start and acquired resistance	Acquired drug resistance category			
	No MDR TB before or after acquiring resistance, no. (%)	Acquired resistance and MDR TB developed later, no. (%)	Initial MDR TB with additional resistance acquired later, no. (%)	Total with acquired drug resistance, no. (%)
<1 mo	20 (36.4)	2 (5.1)	5 (11.9)	27 (19.9)
2 mo	10 (18.2)	0	13 (31.0)	23 (16.9)
3 mo	8 (14.5)	3 (7.7)	4 (9.5)	15 (11.0)
4–6 mo	6 (10.9)	9 (23.1)	10 (23.8)	25 (18.4)
7–9 mo	4 (7.3)	8 (20.5)	5 (11.9)	17 (12.5)
10 mo–1 y	4 (7.3)	7 (18.0)	1 (2.4)	12 (8.8)
>1 y	3 (5.5)	10 (25.6)	4 (9.5)	17 (12.5)
Total	55 (100)	39 (100)	42 (100)	136 (100)

*MDR, multidrug-resistant; TB, tuberculosis.

Most (64.1%, 25/39) TB patients in whom acquired resistance developed into MDR TB had received treatment for >6 months before resistance was acquired (Table 4). Conversely, 76.2% (32/42) of MDR TB patients in whom resistance to additional drugs was acquired had received treatment <6 months before acquiring additional resistance.

### Risk Factors for Acquiring Resistance

Among TB patients with acquired resistance, most were 15–44 years of age (70.3%, 111/158), were male (59.5%, 94/158), were foreign born (75.2%, 115/153), and had pulmonary disease (82.9%, 131/158). A previous TB diagnosis was reported for 23% (32/141). Among those who had acquired resistance >1 year after starting treatment, the demographic profile differed: most were female (58.8%, 10/17) and born in the United Kingdom (66.7%, 5/15), and although they were similar in age, most (58.8%, 10/17) were 15–44 years of age. Among TB patients notified during 2010–2015, a total of 28% (18/65) had >1 social risk factor.

Multivariable model 1 showed that acquired resistance was more likely among TB patients with than without the following characteristics: pulmonary disease (adjusted OR [aOR]  2.1, 95% CI 1.3–3.3); initial resistance to isoniazid (aOR 6.2, 95% CI 3.9–10.0), rifampin (aOR 10.8, 95% CI 3.3–35.3), or both (aOR 41.8, 95% CI 27.0–64.7); and a previous TB episode (aOR 2.3, 95% CI 1.5–3.5) (Table 5). TB patients born in China were 3.4 times more likely (95% CI 1.3–8.8) and those born in South Africa were 2.8 times more likely (95% CI 1.1–7.5) to acquire resistance than those born in the United Kingdom; patients born in India were less likely to acquire resistance than those born in the United Kingdom (aOR 0.4, 95% CI 0.2–0.8). Sensitivity analysis of multivariable model 1, which excluded TB patients who acquired resistance to ethambutol only, pyrazinamide only, or both, showed trends of demographic characteristics that were more or less likely to be associated with acquired resistance similar to those in the original model (Table 6).

**Table 5:** Univariable and multivariable logistic regression model 1 for acquired resistance to ant-TB drugs in England, Wales, and Northern Ireland, 2000–2015*

Characteristic	No acquired drug resistance, no. (%)	Acquired drug resistance, no. (%)	Univariable analysis			Multivariable analysis	
			OR (95% CI)	p value		OR (95% CI)	p value
Age, y							
0–14	1,391 (2.1)	2 (1.3)	1.0	NA		NA	NA
15–44	44,227 (65.5)	111 (70.3)	1.7 (0.4–7.1)	0.4		1.6 (0.4–6.7)	0.5
45–64	12,859 (19.0)	40 (25.3)	2.2 (0.5–9.0)	0.3		2.5 (0.6–10.7)	0.2
>65	9,071 (13.4)	5 (3.2)	0.4 (0.1–2.0)	0.2		0.6 (0.1–3.2)	0.5
Sex							
F	28,453 (42.2)	64 (40.5)	1.0	NA		NA	NA
M	38,985 (57.8)	94 (59.5)	1.1 (0.8–1.5)	0.7		1.2 (0.8–1.6)	0.4
Site of disease							
Extrapulmonary only	24,217 (35.9)	27 (17.1)	1.0	NA		NA	NA
Pulmonary with or without extrapulmonary	43,313 (64.1)	131 (82.9)	2.7 (1.8–4.1)	<0.001		2.1 (1.3–3.3)	0.003
Initial drug resistance							
Drug sensitive	62,721 (92.9)	74 (46.8)	1.0	NA		NA	NA
Isoniazid resistance without MDR	3,830 (5.7)	34 (21.5)	7.5 (5.0–11.3)	<0.001		6.2 (3.9–10.0)	<0.001
Rifampin resistance without MDR	200 (0.3)	3 (1.9)	12.7 (4.0–40.7)	<0.001		10.8 (3.3–35.3)	<0.001
MDR	801 (1.2)	47 (29.7)	49.7 (34.3–72.2)	<0.001		41.8 (27.0–64.7)	<0.001
Country of birth							
United Kingdom	16,184 (26.3)	38 (25.7)	1.0	NA		NA	NA
China	465 (0.8)	6 (4.1)	5.5 (2.3–13.1)	<0.001		3.4 (1.3–8.8)	0.01
India	11,227 1(8.3)	11 (7.4)	0.4 (0.2–0.8)	0.01		0.4 (0.2–0.8)	0.01
Lithuania	238 (0.4)	9 (6.1)	16.1 (7.7–33.7)	<0.001		1.8 (0.8–4.4)	0.2
Pakistan	7,696 (12.5)	15 (10.1)	0.8 (0.5–1.5)	0.6		0.9 (0.4–1.7)	0.6
Somalia	4,184 (6.8)	9 (6.1)	0.9 (0.4–1.9)	0.9		0.9 (0.4–1.9)	0.7
South Africa	759 (1.2)	5 (3.4)	2.8 (1.1–7.1)	0.03		2.8 (1.1–7.5)	0.04
Nigeria	1,324 (2.2)	4 (2.7)	1.3 (0.5–3.6)	0.6		0.8 (0.2–2.6)	0.7
Other	19,417 (31.6)	51 (34.5)	1.1 (0.7–1.7)	0.6		0.9 (0.6–1.5)	0.8
Previous TB episode							
No	54,346 (93.3)	109 (77.3)	1.0	NA		NA	NA
Yes	3,910 (6.7)	32 (22.7)	4.0 (2.7–6.1)	<0.001		2.3 (1.5–3.5)	<0.001

*MDR, multidrug-resistant; NA, not applicable; OR, odds ratio; TB, tuberculosis.

**Table 6:** Sensitivity analysis of univariable and multivariable logistic regression model 1 for acquired resistance to anti-TB drugs in England, Wales, and Northern Ireland, 2000–2015*

Characteristic	No acquired drug resistance, no. (%)		Acquired drug resistance, no. (%)	Univariable analysis			Multivariable analysis	
				OR (95% CI)	p value		aOR (95% CI)	p value
Age, y								
0–14	1,392 (2.1)		1 (0.9)	1.0 (NA)	NA		1.0 (NA)	NA
15–44	44,263 (65.5)		75 (69.4)	2.4 (0.3–17.0)	0.4		2.4 (0.3–17.4)	0.4
45–64	12,871 (19.0)		28 (25.9)	3.0 (0.4–22.3)	0.3		3.0 (0.4–23.2)	0.3
>65	9,072 (13.4)		4 (3.7)	0.6 (0.1–5.5)	0.7		0.9 (0.1–7.8)	0.9
Sex								
F	28,476 (42.2)		41 (38.0)	1.0 (NA)	NA		1.0 (NA)	NA
M	39,012 (57.8)		67 (62.0)	1.2 (0.8–1.8)	0.4		1.4 (0.9–2.1)	0.2
Site of disease								
Extrapulmonary only	24,233 (35.9)		11 (10.2)	1.0 (NA)	NA		1.0 (NA)	NA
Pulmonary with or without extrapulmonary	43,347 (64.1)		97 (89.8)	4.9 (2.6–9.2)	<0.001		3.0 (1.6–5.7)	0.001
Initial drug resistance								
Drug sensitive	62,740 (92.8)		55 (50.9)	1.0 (NA)	NA		1.0 (NA)	NA
Isoniazid resistance without MDR	3,839 (5.7)		25 (23.2)	7.4 (4.6–11.9)	<0.001		6.5 (3.7–11.2)	<0.001
Rifampin resistance without MDR	200 (0.3)		3 (2.8)	17.1 (5.3–55.1)	<0.001		15.2 (4.6–50.2)	<0.001
MDR	823 (1.2)		25 (23.2)	34.7 (21.5–55.9)	<0.001		32.7 (18.9–56.8)	<0.001
Country of birth								
United Kingdom	16,189 (26.3)		33 (33.0)	NA	NA		1.0 (NA)	NA
China	467 (0.8)		4 (4.0)	4.2 (1.5–11.9)	0.01		2.5 (0.8–7.7)	0.1
India	11,232 (18.2)		6 (6.0)	0.3 (0.1–0.6)	0.003		0.2 (0.1–0.6)	0.004
Lithuania	243 (0.4)		4 (4.0)	8.1 (2.8–23.0)	<0.001		0.8 (0.2–2.8)	0.7
Pakistan	7,702 (12.5)		9 (9.0)	0.6 (0.3–1.2)	0.1		0.8 (0.4–1.7)	0.5
Somalia	4,190 (6.8)		3 (3.0)	0.4 (0.1–1.1)	0.1		0.4 (0.1–1.3)	0.1
South Africa	759 (1.2)		5 (5.0)	3.2 (1.3–8.3)	0.02		3.3 (1.2–8.8)	0.02
Nigeria	1,326 (2.2)		2 (2.0)	0.7 (0.2–3.1)	0.7		0.3 (0.04–2.1)	0.2
Other	19,43 (31.6)		34 (34.0	0.9 (0.5–1.4)	0.5		0.7 (0.4–1.2)	0.2
Previous TB episode								
No	54,379 (93.3)		76 (79.2)	1.0 (NA)	NA		1.0 (NA)	NA
Yes	3,922 (6.7)		20 (20.8)	3.6 (2.2–6.0)	<0.001		2.2 (1.3–3.8)	0.01

*aOR, adjusted OR; MDR, multidrug-resistant; NA, not applicable; OR, odds ratio; TB, tuberculosis.

Multivariable model 2, which included social risk factors and strain type lineage, showed that acquisition of drug resistance was more likely among TB patients with than without the following characteristics: age 45–64 years (aOR 1.9, 95% CI 1.0–3.6); pulmonary disease (aOR 2.7, 95% CI 1.2–6.0); and initial resistance to isoniazid (aOR 6.5, 95% CI 2.9–14.4), rifampin (aOR 14.7, 95% CI 1.8–1213.0), or both (aOR 77.3, 95% CI 39.1–152.8) (Table 7). Although a higher proportion of TB patients with (27.7%, 18/65) than without (11.6%, 2,632/22,795) acquired resistance had a social risk factor, after the other factors in model 2 were adjusted for, having a social risk factor was not significantly associated with acquiring resistance. No country of birth was significantly associated with acquired resistance, but TB patients born in India were less likely to acquire resistance than those born in the United Kingdom (aOR 0.3, 95% CI 0.1–0.8). TB patients infected with a Beijing lineage *M. tuberculosis* strain (aOR 3.4, 95% CI 1.6–7.4) or *M. bovis* (aOR 10.3, 95% CI 2.1–49.8) were more likely to acquire drug resistance. Among this subpopulation of TB cases notified during 2010–2015, lineage and country of birth were collinear (p<0.001) and thus not included in the model.

**Table 7:** Univariable and multivariable logistic regression model 2 for acquired resistance to anti-TB drugs in England, Wales, and Northern Ireland, 2010–2015*

Characteristic	No acquired drug resistance, no. (%)	Acquired drug resistance, no. (%)	Univariable analysis			Multivariable analysis	
			OR (95% CI)	p value		aOR (95% CI)	p value
Age, y							
0–14	467 (1.8 )	0	NA	NA		NA	NA
15–44	17,119 (64.6)	48 (64.9)	1.0	NA		1.0	NA
45–64	5,429 (20.5)	23 (31.1)	1.5 (0.9–2.5)	0.1		1.9 (1.0–3 6)	0.04
>65	3,503 (13.2)	3 (4.1)	0.3 (0.1–0.9)	0.05		0.6 (0.2–2.2)	0.5
Sex							
F	10,664 (40.3)	24 (32.4)	1.0	NA		1.0	NA
M	15,820 (59.7)	50 (67.6)	1.4 (0.9–2.3)	0.2		1.3 (0.7–2.3)	0.4
Site of disease							
Extrapulmonary only	10,069 (38.0)	10 (13.5)	1.0 NA	NA		1.0	NA
Pulmonary with or without extrapulmonary	16,443 (62.0)	64 (86.5)	3.9 (2.0–7.6)	<0.001		2.7 (1.2–6.0)	0.01
Initial drug resistance							
Drug sensitive	24,659 (93.0)	25 (33.8)	1.0	NA		1.0	NA
Isoniazid resistance without MDR	1,446 (5.4)	13 (17.6)	8.9 (4.5–17.4)	<0.001		6.5 (2.9–14.4)	<0.001
Rifampin resistance without MDR	50 (0.2)	1 (1.4)	19.7 (2.6–148.4)	0.004		14.7 (1.8–123.0)	0.01
MDR	363 (1.4)	35 (47.3)	95.1 (56.3–160.5)	<0.001		77.3 (39.1–152.8)	<0.001
Country of birth							
United Kingdom	6,239 (24.6)	21 (29.2)	1.0	NA		1.0	NA
China	183 (0.7)	3 (4.2)	4.9 (1.4–16.5)	0.01		2.1 (0.5–9.5)	0.3
India	5,470 (21.6)	5 (6.9)	0.3 (0.1–0.7)	0.01		0.3 (0.1–0.8)	0.02
Lithuania	181 (0.7)	8 (11.1)	13.3 (5.7–30.0)	<0.001		0.6 (0.2–1.8)	0.3
Pakistan	3,270 (12.9)	5 (6.9)	0.5 (0.2–1.2)	0.1		0.7 (0.2–2.5)	0.6
Somalia	1,191 (4.7)	3 (4.2)	0.8 (0.2–2.5)	0.7		0.6 (0.1–2.7)	0.5
South Africa	174 (0.7)	1 (1.4)	1.7 (0.2–12.8)	0.6		1.3 (0.1–11.7)	0.8
Nigeria	579 (2.3)	2 (2.8)	1.0 (0.2–4.4)	0.9		0.9 (0.1–6.9)	0.9
Other	8,080 (31.8)	24 (33.3)	0.9 (0.5–1.6)	0.7		0.7 (0.3–1.4)	0.3
Previous TB episode							
No	23,799 (94.5)	57 (80.3)	1.0	NA		1.0	NA
Yes	1,391 (5.5)	14 (19.7)	4.2 (2.3–7.6)	<0.001		1.7 (0.8–3.4)	0.2
Social risk factors							
0	20,163 (88.4)	47 (72.3)	1.0	NA		1.0	NA
>1	2,632 (11.6)	18 (27.7)	2.9 (1.7–5.1)	<0.001		1.0 (0.5–2.0)	1.0
Organism lineage							
Euro-American	8,631 (39.3)	18 (26.5)	1.0	NA		1.0	NA
Central Asian	6,013 (27.4)	9 (13.2)	0.7 (0.3–1.6)	0.4		1.0 (0.4–2.8)	0.9
East-African-Indian	3,098 (14.1)	6 (8.8)	0.9 (0.4–2.3)	0.9		1.3 (0.5–3.9)	0.6
Beijing	1,230 (5.6)	23 (33.8)	9.0 (4.8–16.7)	<0.001		3.4 (1.6–7.4)	0.002
* Mycobacterium africanum*	192 (0.9)	0	NA	NA		NA	NA
* M.bovis*	124 (0.6)	2 (2.9)	7.7 (1.8-33.7)	0.01		10.3 (2.1–49.8)	0.004
Multiple	599 (2.7)	2 (2.9)	1.6 (0.4–7.0)	0.5		1.4 (0.3–6.7)	0.7
None	2,076 (9.4)	8 (11.8)	1.8 (0.8–4.3)	0.1		1.9 (0.8–4.9)	0.2

*aOR, adjusted OR; MDR, multidrug-resistant; NA, not applicable; OR, odds ratio; TB, tuberculosis.

### Treatment Outcomes

Treatment was completed by a lower proportion of TB patients who did (61.4% [86/140]) than did not (79.9% [45,690/57,209]) acquire drug resistance (p<0.001), and the median duration of treatment was longer among those who did (19.2 months [IQR 12–30 months) than did not (6.2 months [IQR 6–8.8 months]) acquire drug resistance (Table 8). A higher proportion of TB patients with (7.1% [10/140]) than without (4.6% ([2,651/57,209]) acquired resistance died (p<0.001), but time to death was longer for those with acquired resistance (6.8 months [IQR 5.8 months–2.1 years]) than for those without acquired resistance (1.3 months [IQR 0.4–3.2 months]). Among TB patients with acquired resistance, outcomes were worse for those in whom MDR developed than for those with other categories of drug resistance (p = 0.03) (Table 8).

**Table 8:** Treatment outcomes for patients with and without acquired drug resistance to anti-TB drugs, England, Wales, and Northern Ireland, 2001–2013*

Outcomes	Acquired drug resistance category				
	No MDR TB before or after acquiring resistance, no. (%)	Acquired resistance that resulted in MDR TB, no. (%)	Initial MDR TB with additional acquired resistance, no. (%)	Total with acquired drug resistance, no. (%)	No acquired drug resistance, no. (%)
Treatment completed	39 (65)	20 (48.8)	27 (69.2)	86 (61.4)	45,690 (79.9
Died	1 (1.7)	8 (19.5)	1 (2.6)	10 (7.1)	2,651 (4.6)
Lost to follow-up	5 (8.3)	5 (12.2)	3 (7.7)	13 (9.3)	3,046 (5.3)
Still receiving treatment	10 (16.7)	7 (17.1)	5 (12.8)	22 1(5.7)	1,253 (2.2)
Treatment stopped	2 (3.3)	1 (2.4)	3 (7.7)	6 (4.3)	410 (0.7)
Not evaluated	3 (5)	0	0	3 (2.1)	4,159 (7.3)

*MDR, multidrug resistant; TB, tuberculosis.

## Discussion

In England, Wales, and Northern Ireland, acquiring drug resistance while receiving TB treatment is rare (0.2% of cases) but more likely among those with pulmonary disease; with initial resistance to isoniazid, rifampin, or both; who experienced a previous TB episode; or who were born in China or South Africa. Our description of the characteristics of TB patients provides useful insight into the care required for these patients.

For most (57.6%) of the TB patients in our study, either their acquired resistance resulted in MDR TB or they initially had MDR TB and acquired additional resistance, thereby limiting treatment options. Our study demonstrates the value of identifying and preventing acquired drug resistance in patients without MDR TB, one third of whom acquire resistance to isoniazid, one of the main drugs used in standard treatment. A study in China showed that acquired drug resistance among any patients with culture-confirmed TB was much higher (3.7%) than that for our study population (*20*). Studies from India have shown high rates of MDR TB among previously treated patients with drug-sensitive isolates, some of which would be accounted for by acquired resistance to the first-line drugs (*21*).

Most other studies from other low-incidence countries report on TB patients with MDR TB in whom further resistance to second-line drugs is acquired; for example, in the United States, drug resistance was acquired by 5.0% of MDR TB patients, similar to the rate of 5.5% of MDR TB patients identified in our study (*22*–*24*). Numerous studies highlight the need for individualized treatment regimens for patients with MDR TB (*13*,*25*,*26*). In the United Kingdom, the national TB control strategy advocates increasing resources to strengthen a multidisciplinary clinical advisory service that will ensure that MDR TB is treated effectively (*3*). Our study adds to the growing evidence for the need to more closely supervise and monitor MDR TB patients by regular use of DST for second-line drugs and the need to ensure that adequate resources are available to meet patient needs.

In our study, acquired resistance was more likely in TB patients with pulmonary disease and, if ineffectively treated, could lead to the transmission of drug-resistant strains. Another study found that the presence of pulmonary cavitation increased the risk of acquiring resistance (*23*). In our study and others, acquired resistance was more likely in TB patients who had had a previous TB episode, which suggests that these cases were neither successfully treated nor cured during the previous TB episode, most likely because of previous compliance issues (*23*,*27*–*29*). These findings demonstrate the need to efficiently diagnose resistance and ensure that appropriate drug regimens are provided and adhered to.

Our study also found that acquired resistance was more likely in TB patients born in China and South Africa; however, this association was lost when strain lineage was considered. This relationship between country of birth and lineage may be explained by the geographic stratification of *M. tuberculosis* complex lineages. Therefore, the country of birth could instigate greater monitoring of TB treatment for patients in whom acquired resistance is more likely (e.g., those infected with Beijing strains, who acquire MDR TB more readily [*30**,**31*]). Acquired resistance was more likely in patients with TB caused by *M. bovis*; and although the number of patients in our study was small, only 2 patients with acquired resistance had TB caused by *M. bovis*, and both of these patients acquired resistance to isoniazid. This association with *M. bovis* has also been shown by a study in the United States, where *M. bovis* was independently associated with acquiring resistance to isoniazid (*32*).

Our study indicated that resistance was acquired by a higher proportion of TB patients with than without a social risk factor. However, studies in other countries did not consistently find an association between acquired resistance and social risk factors (*7*,*23*,*27*). Our findings indicate that outcomes were poorer (e.g., death or loss to follow-up) among TB patients with than without acquired resistance. Similar results have been reported elsewhere (*9*,*20*,*33*–*35*). Acquisition of resistance occurred later in treatment among patients who acquired resistance that resulted in MDR TB; one quarter acquired resistance >12 months after starting treatment, and their outcomes were worse than those for patients with other types of acquired drug-resistance profiles. Treatment can continue beyond 12 months for several reasons (e.g., initial drug resistance, acquired resistance, adherence issues, or clinical signs that the patient has not been cured), which by international reporting standards would be reported as treatment failed, but in the United Kingdom these patients are reported as still receiving treatment, and outcomes beyond 12 months are reported. Treatment failure has been shown to be associated with late emergence of acquired resistance (*20*), which is consistent with our findings. This evidence illustrates how acquired resistance impedes successful treatment outcomes.

Our study benefits from having a large national cohort followed over an extended period. In the United Kingdom, culture-positive results are routinely matched to case data, enabling the study of drug susceptibility combined with demographic and clinical factors. However, our study does have several limitations. First, some local laboratories may process follow-up cultures without sending the samples on to the *Mycobacterium* reference laboratories, leading to underestimation of acquired resistance. It is also possible that patients with initial resistance (to isoniazid, to rifampin, or MDR TB) may receive more frequent sampling, enabling acquisition of resistance to be more readily detected. Sampling may also differ by site of disease because obtaining multiple samples from patients with extrapulmonary disease may be more difficult. However, these situations are unlikely to occur commonly because failure to respond to treatment would prompt further sample collection. In addition, if there is a delay in collecting samples because of missed/rescheduled appointments, the reported time to diagnosis of acquired resistance may be longer than the actual time to diagnosis.

Although TB guidance in the United Kingdom recommends collecting specimens when TB is suspected, and therefore the initial specimen should be taken before starting treatment, 37% of patients in our study had a treatment start date before the initial specimen collection date. Some of these patients may have acquired resistance before the initial sample was taken, leading to underestimation of acquired resistance. DST results for ethambutol and pyrazinamide often vary between sensitive and resistant because of laboratory and biological artifacts. The effect of this variability is probably minimal because DST was repeated for any isolate that showed resistance to these agents. In addition, our sensitivity analysis, which excluded TB patients who acquired resistance to these agents, showed results consistent with the original model. Last, other factors, such as treatment adherence, may have affected the acquisition of resistance, and those factors associated with acquired resistance identified in this study may not be independent of adherence; however, adherence data are not routinely collected nationally.

Strategies for reducing drug-resistant TB are being implemented in England and globally (*2*,*3*). Whole-genome sequencing has been implemented as a diagnostic tool throughout the United Kingdom, which may bring further insight into the drivers of acquired resistance through identification of mutations (*36*). Whole-gene sequencing will also improve monitoring of acquired resistance to all drugs, first- and second-line, identified for each sample, and will reduce variation in results. The World Health Organization has endorsed a shorter (9-month) treatment regimen for MDR TB, which could potentially improve compliance and reduce acquired resistance (*37*).

These improvements in TB diagnostics and treatment, along with the emphasis placed on reducing MDR TB in national and global strategies, have increased awareness regarding the complex needs of MDR TB patients. Because the likelihood of acquiring resistance is increased among patients with initial drug resistance, these patients have a greater need for more specialized facilities and social services to ensure that appropriate treatment is started quickly and monitored thoroughly. Ongoing monitoring of the level of acquired drug resistance is one indicator of TB control and should be incorporated into routine TB surveillance, especially in countries with higher levels of MDR and acquired resistance (*27*). These results indicate that other countries should undertake similar studies to understand risk factors for acquired drug resistance in their own countries and to ensure that drug resistance is diagnosed and transmission is limited. 
